# Indirect selection of thermal tolerance during experimental evolution of *Drosophila melanogaster*

**DOI:** 10.1002/ece3.1472

**Published:** 2015-04-12

**Authors:** Catriona Condon, Ajjya Acharya, Gregory J Adrian, Alex M Hurliman, David Malekooti, Phivu Nguyen, Maximilian H Zelic, Michael J Angilletta

**Affiliations:** School of Life Sciences, Arizona State UniversityTempe, AZ, 85287

**Keywords:** Chill coma, genetic correlation, knockdown, selection experiment, thermal adaptation, trade-offs

## Abstract

Natural selection alters the distribution of a trait in a population and indirectly alters the distribution of genetically correlated traits. Long-standing models of thermal adaptation assume that trade-offs exist between fitness at different temperatures; however, experimental evolution often fails to reveal such trade-offs. Here, we show that adaptation to benign temperatures in experimental populations of *Drosophila melanogaster* resulted in correlated responses at the boundaries of the thermal niche. Specifically, adaptation to fluctuating temperatures (16–25°C) decreased tolerance of extreme heat. Surprisingly, flies adapted to a constant temperature of 25°C had greater cold tolerance than did flies adapted to other thermal conditions, including a constant temperature of 16°C. As our populations were never exposed to extreme temperatures during selection, divergence of thermal tolerance likely reflects indirect selection of standing genetic variation via linkage or pleiotropy. We found no relationship between heat and cold tolerances in these populations. Our results show that the thermal niche evolves by direct and indirect selection, in ways that are more complicated than assumed by theoretical models.

## Introduction

Theoretical models of thermal adaptation often assume that a trade-off exists between specialists and generalists (Angilletta et al. 2003). If this trade-off exists, an increase in the breadth of the thermal niche would decrease fitness at the mean (Gabriel and Lynch 1992; Gilchrist 1995; Palaima 2007). In other words, a Jack (or Jane) of all temperatures would be a master of none (Huey and Hertz 1984). This specialist–generalist trade-off can be described as a negative genetic correlation between fitness at two or more temperatures (Kingsolver et al. 2001). Because of these genetic correlations, populations that evolve greater mean fitness at one temperature can indirectly lose mean fitness at another temperature (Lande and Arnold 1983).

Selection experiments confirm the presence of genetic correlations by documenting direct and indirect responses to selection. These experiments begin with a set of populations derived from a common source and control the agent of selection over multiple generations. Under controlled environmental conditions, phenotypic divergence between selected and control populations reflects direct selection, a correlated response to direct selection, or genetic drift. Artificial selection experiments have shown that direct selection leads to correlated responses (Hoffmann and Parsons 1993; Partridge and Fowler 1993). In a similar way, natural selection experiments in controlled environments have been used to study the direct and correlated responses to selective agents such as temperature. However, correlated responses do not always support models of specialist–generalist trade-offs (Angilletta 2009). For example, populations of flies (*Drosophila* spp.) adapted to moderate temperatures (14° to 28°C) also acquired greater tolerance of higher temperatures, despite never encountering such extremes during selection (Stephanou and Alahiotis 1983; Quintana and Prevosti 1990; Huey et al. 1991; Cavicchi et al. 1995). These results indicate that a positive genetic correlation can exist between performance in moderate and extreme conditions.

We studied how populations of flies (*Drosophila melanogaster*) tolerated extreme temperatures after adapting to intermediate temperatures. These populations evolved at either a constant temperature (16° or 25°C) or fluctuating temperature (16° and 25°C) for 30–60 generations, leading to physiological and life-historical adaptations (Cooper et al. 2012; Condon et al. 2014). These previous studies found that adaptation of flies to 16° or 25°C was not associated with a loss of performance at other temperatures, as expected from a specialist–generalist trade-off. Although some genotypes might be a jack-of-all-temperatures (Reboud and Bell 1997; Weaver et al. 1999; Hughes et al. 2007; Legros and Koella 2010; Duncan et al. 2011; Ketola et al. 2013; Long et al. 2013; Condon et al. 2014), a more likely explanation is that specialist–generalist trade-offs manifest themselves only at the boundaries of the thermal niche. Therefore, we asked whether populations that had adapted to 16° or 25°C had also diverged in their ability to tolerate extreme temperatures. We hypothesized that flies adapted to moderate temperatures may have reduced thermal tolerance if thermal adaptation is mediated by a generalist–specialist trade-off. As these populations never experienced extreme temperatures, natural selection could target heat or cold tolerance only if this trait were pleiotropically or genetically linked to traits expressed during selection at either 16° or 25°C.

## Materials and Methods

We studied experimentally evolved populations created by exposing five populations of *Drosophila melanogaster* to each of the four thermal conditions (*N* = 20 populations): (1) a constant 16°C (C populations), constant 25°C (H populations), temporal fluctuations between 16° and 25°C (T populations), and spatial variation with migration between 16° and 25°C (S populations). Temporal fluctuations were controlled by moving the T populations between rooms at 16° and 25°C every 4 weeks. Spatial variation was maintained by subdividing each population and keeping half of the population at 16°C and the other half at 25°C; eggs were manually transferred between divisions every 4 weeks. To control for this population, cages for the other conditions were also subdivided but both divisions experienced the same temperature(s). The photoperiod was maintained at 12:12 h for all populations. These populations were sampled in 2009, when those at 16° and 25°C had completed 32 and 64 generations, respectively; an intermediate number had occurred in populations exposed to fluctuating temperature. To preserve the genetic diversity within and among populations for future studies, in August 2009, isofemale lines were founded from by pairing virgin flies from each population. Genetic correlations can be calculated by measuring traits on different flies from an inbred isofemale line. However, these lines can also suffer low fecundity, genetic drift, and other issues due to long-term laboratory maintenance (David et al. 2005). The flies used in this study were derived from a single population in British Columbia, Canada. Additional information about the origin of the experimental populations and the isofemale lines can be found elsewhere (Yeaman et al. 2010; Condon et al. 2014).

Our experiments included a subset of the original 400 isofemale lines created from the 20 selective populations. Several of the 400 isofemale lines created in 2009 had gone extinct, and others were excluded from the thermal tolerance assays due to low or zero fecundity after two generations of density control. In June 2012, we controlled the density of each isofemale line for two generations by transferring only two adults of each sex into new vials to lay for 48 hrs. Following this period of density control, pairs of 7-day-old females from each isofemale line were transferred to fresh vials. These vials were kept at 20.5°C, which is intermediate to the temperatures used in the selection experiment. After 48 h, females were removed to limit the density of offspring in each vial. The vials were kept at 20.5°C until the offspring emerged as adults. These adults were used in our studies of thermal tolerance. Throughout these experiments, isofemale lines were maintained in 25 × 90 mm vials (Genesee Scientific, San Diego, CA) on ∼3–4 cm of the Bloomington Standard corn meal–corn syrup diet.

We tested the heat tolerance of male and female flies from the selective populations by examining the time until knockdown at 39.5°C. Newly emerged flies from each density-controlled isofemale line were separated by sex and placed onto new food. Between 7 and 10 days after emergence, one male and one female per line were each transferred without anesthesia into individual 10-mL glass vials with a stopper. Files were kept in vials for only a short time (<5 min) before tolerance was measured. We used two custom-built knockdown chambers that consisted of a clear acrylic box (28 × 4 × 7.5 cm) with a sealed, watertight lid. Inflow and outflow valves were drilled into the short side of each chamber, and water at a constant temperature (39.5 ± 0.1°C) was continually flushed though from a controlled water bath (VWR, Radnor, PA. 11505). Eight capped 10-mL vials containing individual flies were fitted into milled holes in the lid of the chamber and were completely submerged in water when the chamber was sealed. In each trial, the time that each fly collapsed was recorded as the knockdown time (Huey et al. 1992). Each trial lasted until a time was recorded for all eight flies with the aid of the software JWatcher (Macquarie University, Australia). We recorded the knockdown time at 39.5 ± 0.1°C for 306 female and 304 male flies. Knockdown time was recorded over 3 days on 84, 81, 91, and 85 isofemale lines from the C, H, S, and T selection environments, respectively.

To examine the cold tolerance of the selective populations, we reared adult flies under the same density control conditions as mentioned above. To begin the chill coma, we transferred adult males and females between 7 and 10 days old into empty narrow fly vials with stoppers and placed them into an ice slurry for 16 h. Isofemale lines were randomly distributed among four temporal blocks for chilling. After 16 h, vials were removed from the ice and flies were placed on a sheet of paper at room temperature (∼21°C). The time until recovery from the chill coma was scored for all flies. Recovery time was scored to the nearest second when a fly had successfully righted itself and began to walk. Any flies that had not recovered after 75 min was scored as censored data in our survival analysis. Chill coma recovery was recorded on 534 individuals (female *N *= 268, male *N* = 266), from 274 isofemale lines. We recorded chill coma recovery from 151, 107, 133, and 143 individuals from C, H, S, and T selection environments, respectively.

As thermal tolerance of a fly can depend on its size, we measured the wing size of 434 males and females from 213 isofemale lines to use as a covariate in our statistical analyses. The procedure for measuring wing size followed that of Condon et al. (2014). Twelve landmarks from the left wing (see Fig.1 of Yeaman et al. 2010) were digitized using a software package, TpsDIG2 (Rohlf 2001). To estimate wing area, we used the sum of the squared coordinates of the 12 landmarks, referred to as centroid size (Hoffmann and Shirriffs 2002). To analyze sources of variation in wing area, we fit a linear model according to Zuur et al. (2009). Population nested within selective environment was fit as a random effect. Sex and selective environment were fixed effects. Parameter estimation and model selection were performed according to Zuur et al. (2009). Contrasts of the marginal means were used to examine differences among the selective environments with the Holm method used to adjust *P* values for multiple comparisons.

**Figure 1:**
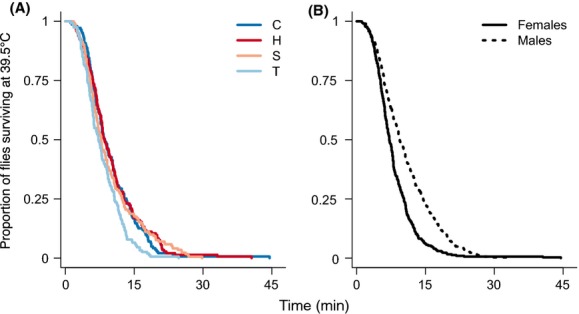
Survival time at 39.5°C for populations of *Drosophila melanogaster* experimentally evolved for 3 years at constant and fluctuating thermal environments. (A) Knockdown time for populations evolved in either a constant 16°C (C populations), a constant 25°C (H populations), a temporally fluctuating (T populations), or spatially fluctuating (S populations) thermal condition. (B) Knockdown time for male and female *D. melanogaster* pooled across all selective populations.

Thermal tolerances were analyzed using Cox proportional hazard models. After 75 min, 118 of 534 flies failed to recover from chill coma; these individuals were censored in the analysis. As all flies succumbed to heat exposure, no individuals were censored in the analysis of knockdown time. Sex and selective environment (H, C, T or S) were fixed effects, and wing size was a covariate. Population nested within selective environment and temporal block were fit as a random effects. All models were fit using the *coxme* (Therneau 2012) and *lme4* libraries in R (V.3.0.2, R Core Team, 2013). Significance of the fixed effects during model selection was determined using likelihood ratio tests. For post hoc tests, parameter estimates of interest were selected for further investigation and compared using contrasts with adjusted *P* values (Holm). We used the *multcomp* (Hothorn et al. 2008) package in R to perform all post hoc analyses.

We fit a linear mixed effect model to test for a genetic correlation between thermal tolerances within each isofemale line. Correlations among traits within isofemale lines can be interpreted as estimates of broad-sense genetic correlations. Knockdown time was the dependent variable, and chill coma recovery, selective environment, size, and sex were fit as explanatory variables. Continuous variables were rescaled to center around zero. Population within selection environment and isofemale line were included as random effects. The best-fit model was determined via AIC. We also examined whether female thermal tolerance data were correlated with the fecundity of these lines measured in an earlier study (Condon et al. 2014). Fecundity was the total number of eggs produced by 25°C developing females from an isofemale line at all seven temperatures tested in that study. We fit a linear mixed effect model using the lme4 package in R. Selective environment, size, recovery time, and knockdown time were fit as fixed effects. Population within selective environment was included as a random effect. Significance of the fixed effects was determined using Wald’s Type III chi-square tests. For all analyses, significance was taken at the level *P* < 0.05).

## Results

### Heat tolerance

Populations that evolved in a temporally fluctuating environment (T populations) diverged in heat tolerance from those that evolved in all other selective environments. Specifically, experimental evolution at fluctuations between 16° and 25°C caused the evolution of reduced heat tolerance, such that flies from these populations succumbed faster when exposed to 39.5°C (*χ*^2^ = 9.77, df = 3, *P *= 0.02, Fig.1A). The median heat tolerance at 39.5°C for T population flies was 7.2 min, while the median knockdown times from the S, H, and C populations were 7.8, 8.5, and 8.6 min, respectively. Post hoc comparisons revealed that knockdown times of flies from the T populations differed significantly from those of all other populations: T vs. S (*Z *= 2.4, *P *= 0.04), T vs. C (*Z *= 2.6, *P *= 0.03), and T vs. H (*Z *= 3.0, *P *< 0.01). The variation among populations could not be explained by size, because wing area was omitted from the most likely statistical model (likelihood ratio test: *χ*^2^* *= 0.25, df* *= 1, *P *= 0.6). Additionally, the divergence of T populations was unlikely to have resulted from genetic drift; the random effect of population improved neither the fit of the random component of the model (LRT: *χ*^2^* *= 0.73, df* *= 2, *P *= 0.6) nor the fit of the overall model (LRT: *χ*^2^* *= 0.01, df* *= 2, *P *= 0.9).

### Cold tolerance

**Figure 2:**
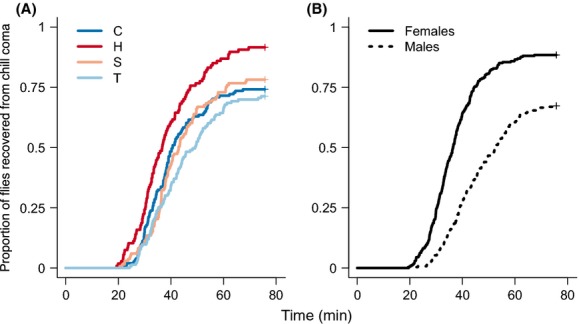
Recovery time from 16 h chill coma of *Drosophila melanogaster* experimentally evolved in constant and fluctuating thermal environments. (A) Chill coma recovery time of flies evolved in either a constant 16°C (C populations), a constant 25°C (H populations), temporally (T populations), or spatially (S populations) fluctuating thermal condition. (B) Chill coma recovery time for males and female *D. melanogaster* pooled across all selective populations.

A surprising pattern of cold tolerance was observed among populations. Flies that evolved at a constant 25°C (H populations) recovered from chill coma more rapidly than did flies from any other selective environment (Fig.2A): H vs. T (*Z *= 3.71, *P <* 0.01), H vs. S (*Z *= 2.65, *P *= 0.04), and H vs. C (*Z *= 2.84, *P *= 0.02). The variation among populations seems easier to digest when described as percentages rather than times. After 75 min, 92% of flies had recovered from chill coma in the H populations, whereas only 75%, 72%, and 78% of flies had recovered in the C, T, and S populations, respectively. This unexpected increase in cold tolerance in H populations probably resulted from indirect selection rather than genetic drift. A random effect of population nested within selective environment only marginally improved the fit of the statistical model (*χ*^2^* *= 5.3, df* *= 1, *P *= 0.07). Among all 20 populations, only two had 100% recovery from chill coma after 75 min, and both of these were H populations. Finally, the variation in recovery time among H populations was smaller than among C, T, or S populations.

Divergence in cold tolerance cannot be attributed to a divergence in body size among populations. We found no significant difference among populations in the wing areas of flies, which is a common index of body size (*χ*^2^* *= 5.5, df* *= 3, *P *= 0.2). Even when we used post hoc analyses to compare wing areas of flies in H populations and those of flies in each of the other types of populations, we found no significant differences (Tukey’s HSD: *P *> 0.1 for all comparisons).

### Sex effect in thermal tolerance

Both knockdown times and chill coma recovery were sexually dimorphic (Figs.1B, 2B). Males resisted exposure to heat longer (*N *= 610*, Z *= 6.26, *P *< 0.001) but recovered from chill coma more slowly (*N *= 534, *Z *= 9.7, *P *< 0.001) than females did. The median knockdown time for males (9.4 min) was more than 2 min longer than that of females (7.1 min). The median time for females to recover from chill coma (36.4 min) was 16 min faster than that of males (52.7 min). Although mean wing area of females was 11% greater than that of males *(T *= 28.7, *P *< 0.001, *N *= 434), the poor relationship between wing area and thermal tolerance suggests that sexual dimorphism of body size cannot explain sexual dimorphism of thermal tolerances.

### Genetic correlations between traits

Chill coma recovery did not explain significant variation in knockdown (*χ*^2^* *= 0.1, df* *= 1, *P *= 0.9), indicating no correlation exists between these thermal tolerance traits. Additionally, we used data from a previous experiment to see whether thermal tolerances of females were correlated with daily fecundity (Condon et al. 2014). We found that heat tolerance did not contribute significant variation in fecundity. By contrast, cold tolerance might explain some proportion of daily fecundity, as indicated by a marginally significant interaction between cold tolerance and selective population (*χ*^2^* *= 7.68, df* *= 3, *P *= 0.05).

## Discussion

We hypothesized that populations of flies adapting to 16° or 25°C would lose some ability to tolerate more extreme temperatures, a trade-off potentially mediated by pleiotropy or linkage. Although we observed evidence of indirect selection, responses were uncorrelated with daily fecundity, a trait that diverged through direct selection. Overall, flies in populations that evolved at fluctuating temperature had greater daily fecundity (Condon et al. 2014) but succumbed more rapidly extreme heat (this study) than did flies in populations that evolved at either 16° or 25°C. Similarly, flies in populations that evolved at 25°C recovered from chill coma more rapidly than did flies in other populations, including those populations that evolved at 16°C. Nevertheless, neither knockdown time nor recovery time covaried with daily fecundity among populations. Thus, abilities to tolerate extreme temperatures evolved independently of fecundity at intermediate temperatures.

As our populations were never exposed to extreme heat or cold, thermal tolerances must have evolved through a pleiotropic allele, a linked gene, or genetic drift. Genetic drift seems the least probable explanation, because the random effect of population nested in selective environment explained little variation in our statistical models. Thus, the divergence of heat and cold tolerances probably reflect indirect selection of standing genetic variation, genetic linkage, or by a single gene that affects multiple traits (pleiotropy). In either case, the poorer heat tolerance of T populations and the better cold tolerance of H populations would have resulted from positive selection of an allele that enhanced performance at 16° or 25°C. As temperature influences the evolution of body size (Partridge et al. 1995), selection for size could have altered heat or cold tolerance; however, wing sizes of flies did not diverge among selective environments. Genes that influence fecundity are an unlikely source of pleiotropy or linkage because we found no significant correlation between fecundity and tolerance. Indirect selection of thermal tolerances could have resulted from any other genes involved in thermal adaptation. For example, earlier work found that our T populations also evolved a greater capacity to regulate the fluidity of cellular membranes, when compared to our H and C populations (Cooper et al. 2012). Studies of other experimental populations uncovered genetic correlations between thermal tolerance and traits expressed in either stressful or benign environments. Artificial selection for greater cold tolerance has impacted longevity (Anderson et al. 2005), starvation resistance (Hoffmann et al. 2005), desiccation (Bubliy and Loeschcke 2005; Sinclair et al. 2007), and fecundity (Hoffmann and Parsons 1989; Watson and Hoffmann 1996). Likewise, populations selected for desiccation resistance have evolved reduced cold tolerance (Hoffmann and Parsons 1989; Sinclair et al. 2007).

Heat tolerance in T populations might have been impacted by indirect selection of a deleterious allele. Cryptic genetic variation – genetic variation expressed in environments that are rarely encountered – enables mutations to accumulate at loci under relaxed selection (Paaby and Rockman 2014). As the heat tolerance of T populations was better explained by a treatment effect than a random effect of population nested within selection environment, we doubt that these populations independently acquired a deleterious mutation. However, relaxed selection of heat tolerance could have enabled a cryptic deleterious allele to hitchhike to fixation, if this allele was physically linked with an allele that improved performance at 16° or 25°C. Because heat tolerance did not diverge between the constant (C and H populations) and the spatially variable (S population) selective environments, the beneficial allele must have been selectively neutral in these environments.

We found no evidence that heat and cold tolerances were genetically correlated in our populations, which accords with evidence from other selection experiments and natural populations (Hercus et al. 2000; Anderson et al. 2005; Udaka et al. 2010; Nyamukondiwa et al. 2011). The failure to detect a genetic correlation could reflect the methods used to assess thermal tolerance more than the genetic basis of thermal tolerance (Rezende et al. 2014). Still, at least one experiment with *D. melanogaster* documented a genetic correlation between heat and cold tolerances; specifically, selection for rapid recovery from chill coma led to rapid recovery from heat coma as well (Mori and Kimura 2008). These positive correlations involve alleles that improve recovery from stressful conditions, whether hot or cold. For example, heat-shock proteins help insects recover from extreme temperatures as well as other stresses, such as desiccation (Rinehart et al. 2007; Colinet et al. 2010). However, these proteins are down-regulated at benign temperatures to avoid deleterious impacts on growth and development (Feder et al. 1992; Hoffmann 1995). Thus, direct selection at 25°C was unlikely to boost concentrations of heat-shock proteins in flies, making the rapid recovery from chill coma by flies in H populations surprising.

Our results support the view that natural selection at moderate temperatures can cause the correlated evolution of traits expressed at extreme temperatures. However, these correlated responses do not necessarily reflect trade-offs between performances at moderate and extreme temperatures, suggesting that some genotypes have broader thermal niches without paying an obvious cost (Reboud and Bell 1997; Weaver et al. 1999; Hughes et al. 2007; Legros and Koella 2010; Duncan et al. 2011; Ketola et al. 2013; Long et al. 2013; Condon et al. 2014). Trade-offs associated with thermal adaptation can be difficult to detect when only a few traits are measured. Widening the phenotypic focus to including other environmental dimensions of the niche should help to identify the reasons why generalists do not evolve under all conditions. As genetic correlations can weaken and even reverse within selection experiments (Archer et al. 2003; Chippindale et al. 2003; Phelan et al. 2003), many experiments like ours must accumulate before general picture of the cost of adaptation will emerge.
